# Platelet Transfusion in Patients With Sepsis and Thrombocytopenia: A Propensity Score-Matched Analysis Using a Large ICU Database

**DOI:** 10.3389/fmed.2022.830177

**Published:** 2022-02-16

**Authors:** Shuangjun He, Chenyu Fan, Jun Ma, Chao Tang, Yi Chen

**Affiliations:** Department of Emergency, Renji Hospital, Shanghai Jiao Tong University School of Medicine, Shanghai, China

**Keywords:** sepsis, thrombocytopenia, platelet transfusion, mortality, ICU—intensive care unit

## Abstract

**Purpose:**

Sepsis with thrombocytopenia is highly prevalent in critically ill intensive care unit (ICU) patients and is associated with adverse outcomes. Platelet transfusion is the primary treatment of choice. However, evidence for the beneficial effects of platelet transfusion in patients with sepsis and thrombocytopenia is scarce and low in quality. This study aimed to evaluate the association between platelet transfusion and mortality among ICU patients with sepsis and thrombocytopenia.

**Patients and Methods:**

Using the Medical Information Mart for Intensive Care III database (v. 1.4), the outcomes of sepsis patients with platelet counts of ≤ 150,000/μL were compared between those who did and did not receive platelet transfusion. The primary outcomes were 28- and 90-day all-cause mortalities. The secondary outcomes were red blood cell (RBC) transfusion, ICU-free days, and hospital-free days. Propensity score matching was employed to assemble a cohort of patients with similar baseline characteristics.

**Results:**

Among 7,765 eligible patients, 677 received platelet transfusion and were matched with 677 patients who did not receive platelet transfusion according to propensity scores. Platelet transfusion, as compared with no platelet transfusion, was associated with an increased risk of 28-day all-cause mortality [36.9 vs. 30.4%, odds ratio (OR), 1.21; 95% confidence interval (CI), 1.01–1.46; *p* = 0.039], increased risk of 90-day all-cause mortality (50.8 vs. 44.6%, OR, 1.13; 95% CI, 1.00–1.31; *p* = 0.048), fewer mean (standard deviation) 28-day ICU-free days (15.88 ± 8.97 vs. 18.64 ± 8.33 days, *p* < 0.001), and fewer hospital-free days (10.29 ± 8.49 vs. 11.43 ± 8.85 days, *p* = 0.017). The rate of RBC transfusion was not significantly different between the platelet transfusion and non-transfusion groups (*p* = 0.149). The results were maintained across several subgroup and sensitivity analyses.

**Conclusion:**

In this study, platelet transfusion was associated with higher 28- and 90-day all-cause mortalities. These results suggest the potential hazards of platelet transfusion in ICU patients with sepsis and thrombocytopenia.

## Introduction

Despite considerable improvements in the management of sepsis, it remains a global public health challenge (1). The incidence of sepsis is increasing at a rate of 8–13% annually; sepsis is a leading cause of long-term disability and is responsible for ~15 million annual deaths (2). Sepsis is defined as a state of multiple organ dysfunction caused by a dysregulated host response to infection. Among those responses, thrombocytopenia is a major complication of sepsis, present in 55% of cases, and correlates with a poor prognosis (3).

Platelet transfusion are commonly used in bone marrow transplant patients, in oncology patients receiving chemotherapy, and to prevent bleeding before invasive procedures in patients with thrombocytopenia. Around 3–15% of sepsis patients with thrombocytopenia receive platelet transfusion in various intensive care unit (ICU) settings. Pre-transfusion platelet counts vary widely from 10,000 to 150,000/μL, depending on the indication for transfusion (4). However, the most recent Surviving Sepsis Campaign guidelines on treating patients with thrombocytopenia address this topic in a vague and limited way (1). For sepsis with thrombocytopenia, there is no evidence for the use of platelet transfusion. Apart from expert opinion and several single-center retrospective studies, no data exist on key clinical outcomes including bleeding, days spent in the hospital, and death (5, 6). Meanwhile, the benefits of platelet transfusion in sepsis patients with thrombocytopenia have been questioned (7, 8). Additionally, several studies suggested that platelet transfusion is associated with adverse effects including acute myocardial infarction, infection, stroke, thrombosis, and lung injury (9–13).

Therefore, there is considerable uncertainty regarding the benefits of platelet transfusion. To address this uncertainty, we aimed to examine the relationship between platelet transfusion and prognosis in patients with sepsis and thrombocytopenia, using data from a large critical care database.

## Materials and Methods

### Study Design

We conducted a retrospective cohort study based on a large US-based database called the Medical Information Mart for Intensive Care III (MIMIC-III). The MIMIC-III (v1.4) database includes data on 53,423 distinct ICU admissions for patients at the Beth Israel Deaconess Medical Center between 2001 and 2012 (14). One author (Chenyu Fan) obtained access to the database and was responsible for data extraction (certification number 27252652). Informed consent was not obtained because the data were obtained from publicly available sources.

### Selection of Participants

Patients aged 18 years or older who fulfilled the criteria for sepsis combined with thrombocytopenia were eligible for inclusion. Sepsis was diagnosed according to the Sepsis-3 criteria; specifically, if patients presented with documented or suspected infection and an acute change in the total Sequential Organ Failure Assessment (SOFA) score of ≥2 points, they were considered to have sepsis (1). Thrombocytopenia was defined as any platelet count ≤ 150,000/μL. Patients were excluded if they were pregnant or breastfeeding; died within 48 h of admission to ICU; had active hematological or autoimmune disorders; had a discharge diagnosis of thrombotic thrombocytopenic purpura (TTP), hemolytic uremic syndrome (HUS), or heparin-induced thrombocytopenia (HIT); and had received a red blood cell (RBC) transfusion prior to platelet transfusion or showed a decrease in the hemoglobin level of more than 20 g/L. For multiple admissions of the same patient, we used the initial record.

### Variable Extraction

We retrieved the medical records of sepsis patients with thrombocytopenia. Information on the following parameters was extracted: baseline characteristics within 24 h of admission to the ICU, sex, race, comorbidities, medication history, infection site (respiratory, urinary, gastrointestinal, and others), ICU type (medical or surgical), admission period, severity at admission measured by the SOFA score, quick SOFA (qSOFA) score, mechanical ventilation use, renal replacement therapy use, administration of vasopressors, RBC transfusion events and the time-points of RBC transfusion, and vital status. Comorbidities including diabetes mellitus, renal failure, liver disease, and coagulopathy were identified on the basis of the recorded International Classification of Diseases, Ninth Revision codes. For medication history, we reviewed medications that could affect platelet function or cause abnormal coagulation test results (e.g., aspirin, clopidogrel, and warfarin).

The exposure factor evaluated in our study was platelet transfusion. If the patient did not receive a transfusion, the lowest platelet count during the ICU stay was analyzed. If the patient received a platelet transfusion, the lowest platelet count before transfusion was used. For each platelet unit administered, we recorded the date of transfusion, the dose, and the time from platelet nadir to transfusion. If the patient received multiple transfusion, analysis was limited to the first platelet transfusion. Given that the majority of thrombocytopenia events occur within 10 days of sepsis onset, we selected 2 weeks as the cutoff time for transfusion (15), which would also control for other common causes of thrombocytopenia in ICU patients.

### Outcomes

The primary outcomes measured were 28- and 90-day all-cause mortalities. The secondary outcomes were RBC transfusion, ICU-free days, and hospital-free days. ICU-free days were defined as the number of days between the day of ICU discharge and day 28 after study enrollment. This was defined as 0 if the patient died before 28 days or if the patient remained in the ICU for more than 28 days. We defined hospital-free days as the number of days between the day of hospital discharge and day 28 after ICU admission. RBC transfusion was considered as a secondary outcome where the transfusion had to be administered after the platelet transfusion (in patients who had a platelet transfusion, but not for patients who did not have a platelet transfusion).

### Statistical Analyses

We used a 1:1 propensity score-matching method without replacement to match sepsis-associated thrombocytopenia patients with similar baseline characteristics between the platelet transfusion and non-platelet transfusion groups. The caliper width was set to 0.25 standard deviations (SDs) of the logit propensity score. To assess pre- and post-match imbalances, standardized differences were estimated for all baseline covariates. A standardized difference of <10% was considered to indicate an adequate balance.

The end points of 28-day all-cause mortality, 90-day all-cause mortality, and RBC transfusion were modeled using a conditional logistic regression. The endpoints of ICU- and hospital-free days were analyzed using paired student-t test.

The supplementary table showed the differences in the patients with missing lactate and patients without missing lactate (see Supplementary Table 1). Missing values of lactate were processed by two strategies: (1) conditional mean interpolation, and (2) conversion of the lactate variable into five possible categorical variables including (0.2), (2.4), (4.10), >10, and missing values. The area under the receiver operating characteristic curve (AUC) was the main factor used to evaluate the model. The Akaike information criterion (AIC), Bayesian information criterion, and pseudo R-squared values were also used to compare different possible models and determine which one had the best fit. The model including lactate showed better performance than not containing (AUC 0.8416 vs. 0.8395; AIC 3642.44 vs. 3656.84; Pseudo R-squared 0.27 vs. 0.26; Supplementary Table 2).

To perform 1:1 propensity score matching, we estimated each patient's propensity to receive a platelet transfusion, using a logistic regression model. To build this model, we initially identified candidate variables [sex, age, race, ICU type, infection site, platelet count, qSOFA score, SOFA score, diabetes mellitus, renal failure, liver disease, coagulopathy, renal replacement therapy, mechanical ventilation, vasopressor use, hemoglobin, lactate, international normalized ratio (INR), creatinine, antiplatelet drug use, and warfarin use] on the basis of their prior possibility of confounding the relationship between platelet transfusion and our chosen end points (see Supplementary Figure 1). We then used Hosmer and Lemeshow's purposeful selection approach to determine the reserve variables (16).

Three sensitivity analyses were employed for the primary outcomes: the first used a multivariable model for the full cohort adjusting for all variables used in propensity score matching; the second replaced the missing lactate values with the mean lactate values, and the third only included patients who received platelet transfusion within 1 day of platelet nadir.

All statistical analyses were performed using R version 4.0.3 (R foundation for Statistical Computing, Vienna, Australia). We used R's MatchIt package to carry out propensity score matching (17).

## Results

**Figure 1:**
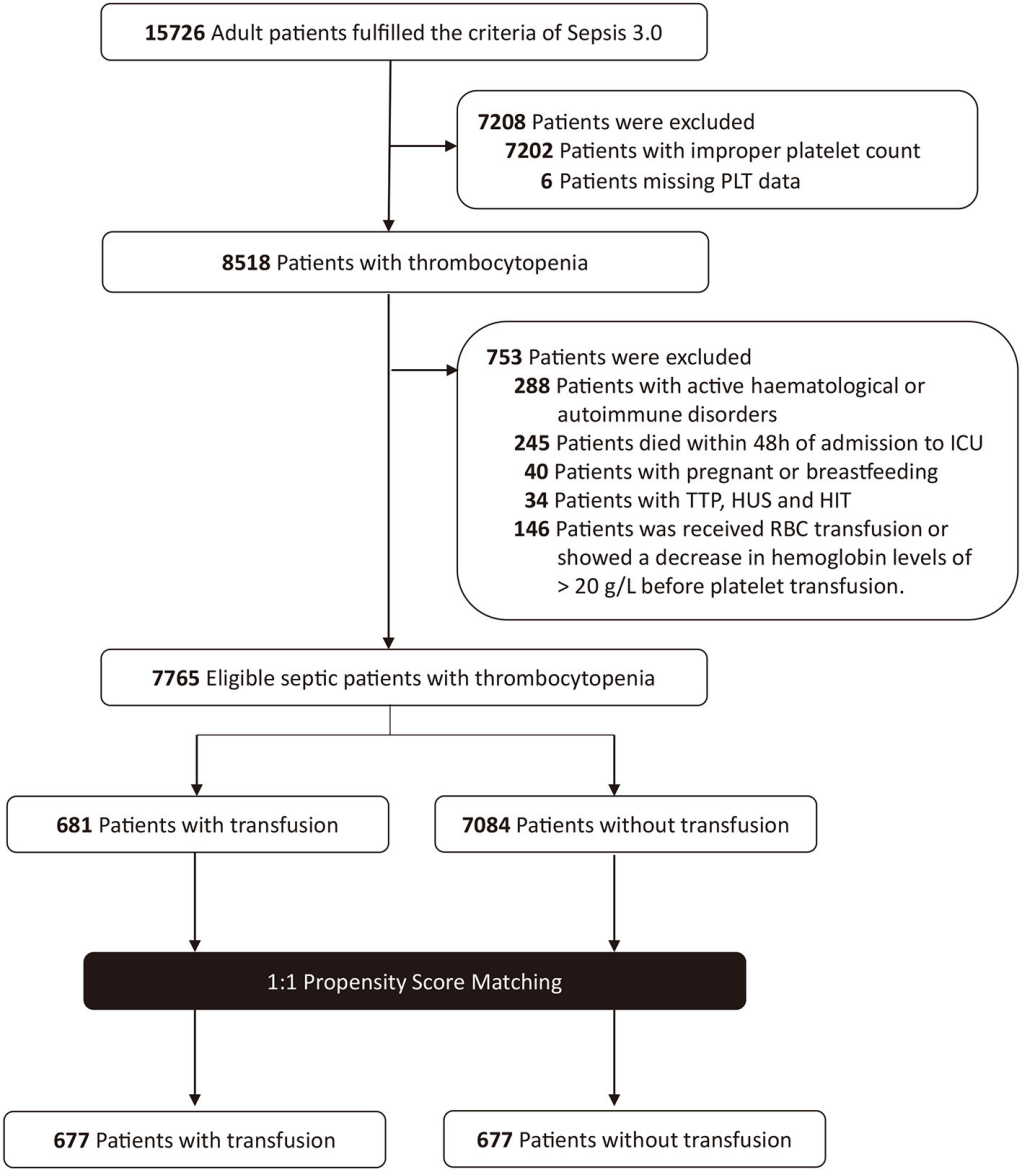
Study population flow diagram. PLT, platelet; TTP, thrombotic thrombocytopenic purpura; HUS, hemolytic uremic syndrome; HIT, heparin-induced thrombocytopenia; RBC, red blood cell.

**Figure 2:**
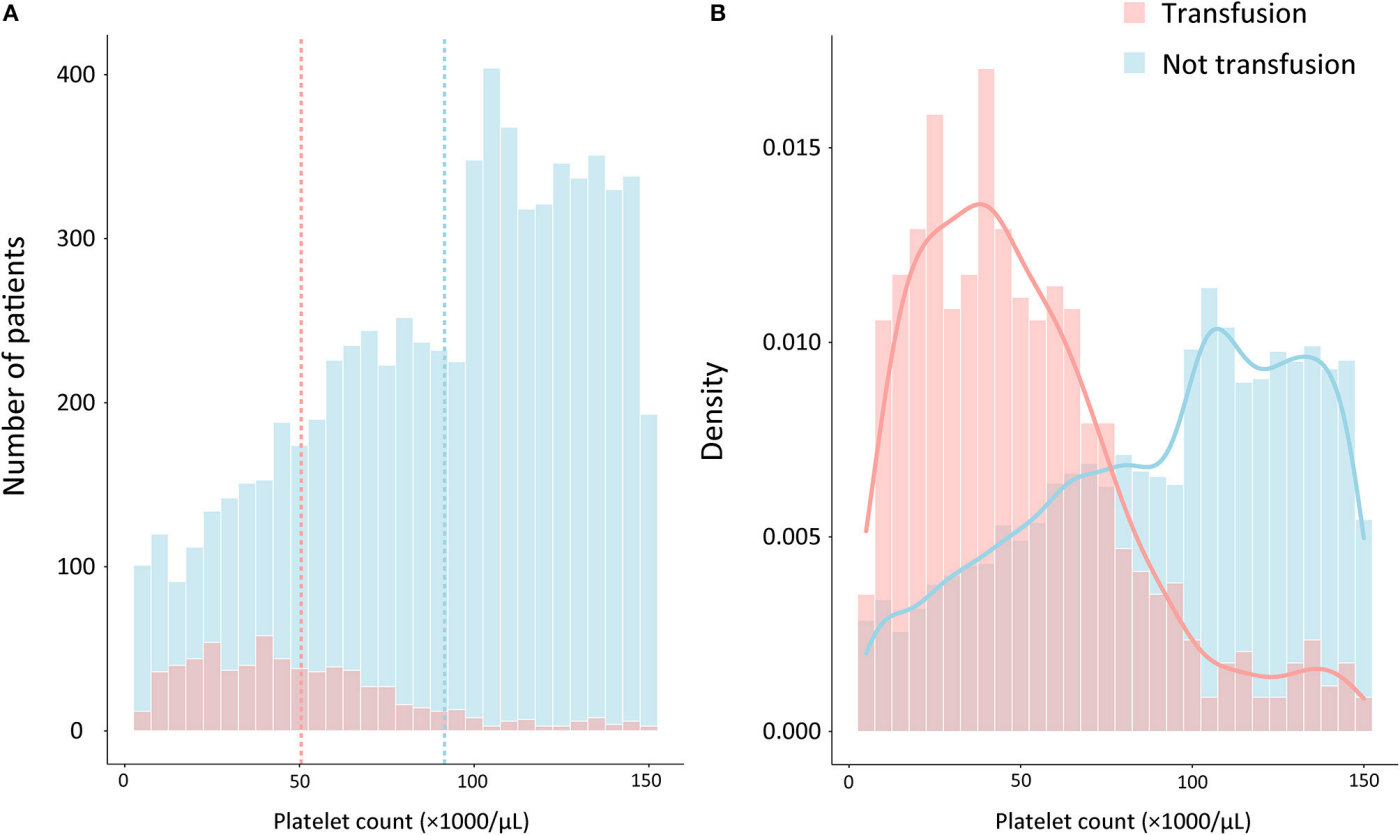
Distribution of patients by platelet counts and the proportion of platelet transfusion on admission among septic patients with thrombocytopenia. **(A)** The frequency distribution of the number of platelet counts, with the vertical dashed lines representing the mean platelet count for transfusion group (red) and not transfusion group (blue), respectively. **(B)** Probability density distributions of platelet counts for transfusion group (red curves) and not transfusion group (blue curves).

A total of 15,726 patients who met the Sepsis-3 criteria were admitted between 2001 and 2012. Patients were excluded if they had improper platelet counts (*n* = 7,202); had active hematological or autoimmune disorders (*n* = 288); died within 48 h of admission to ICU (*n* = 245); were pregnant or breastfeeding (*n* = 40); or had TTP, HUS, or HIT (*n* = 34). Altogether, 146 patients who had received an RBC transfusion before the platelet transfusion or showed a decrease in hemoglobin levels of >20 g/L were also excluded. Eventually, a total of 7,765 patients met the criteria. Among these, 681 patients were administered platelet transfusion, and 7,084 patients were not (Figure 1). The distributions of platelet count and density among patients who received a transfusion and those who did not are shown in Figure 2.

Before propensity-score matching, there were differences between the two groups in several of the baseline characteristics (Table 1). Patients who received platelet transfusion were younger, had more comorbidities, and had more severe disease (as determined by the SOFA score). With the use of propensity-score matching, 6,407 non-transfused and four transfused patients were excluded, leaving a matched cohort of 677 patients in each group. The C-statistic for the model presented in Supplementary Figure 2 was 0.8416. The details before and after matching were summarized in Supplementary Figure 3 and the standardized differences were <10.0% for all variables, indicating only small differences between the two groups. The transfusion mainly occurred within 1 day of the lowest platelet measurement or the day of eligible for inclusion (Supplementary Figure 4) and the most of transfused patients received two units of platelets during their hospital stay (Supplementary Figure 5).

**Table 1:** Baseline characteristics of patients in the full cohort and propensity score matched groups.

**Covariate**	**Full cohort**				**Matched cohort**			
	**Not Transfusion** **(*n* = 7084)**	**Transfusion** **(*n* = 681)**	**SMD (%)**	***P*** **-value**	**Not transfusion** **(*n* = 677)**	**Transfusion** **(*n* = 677)**	**SMD (%)**	***P*** **-value**
Male, *n* (%)	4,077 (57.60)	405 (59.50)	3.9	0.333	382 (56.40)	402 (59.40)	6.0	0.271
Age (years)	66.83 ± 15.87	62.02 ± 15.89	30.2	<0.001	62.04 ± 16.21	62.00 ± 15.88	0.3	0.963
White race, *n* (%)	5,130 (72.40)	489 (71.80)	1.4	0.734	489 (72.20)	485 (71.60)	1.3	0.809
ICU type, *n* (%)								
SICU	2,555 (36.10)	324 (47.60)	23.5	<0.001	300 (44.30)	321 (47.40)	6.2	0.252
MICU	4,529 (63.90)	357 (52.40)	23.5	<0.001	377 (55.60)	356 (52.60)	6.2	0.252
Comorbidity								
Diabetes mellitus, *n* (%)	2,124 (30.00)	127 (18.60)	26.7	<0.001	131 (19.40)	126 (18.60)	1.9	0.729
Renal failure, *n* (%)	1,704 (24.10)	78 (11.50)	33.4	<0.001	74 (10.90)	78 (11.50)	1.9	0.731
Liver disease, *n* (%)	950 (13.40)	152 (22.30)	23.4	<0.001	157 (23.20)	152 (22.50)	1.8	0.746
Coagulopathy, *n* (%)	771 (10.90)	190 (27.90)	44.1	<0.001	177 (26.10)	188 (27.80)	3.7	0.501
Laboratory examination								
Platelet count (×1,000/μL)	91.54 ± 39.09	50.50 ± 31.50	115.6	<0.001	48.11 ± 32.26)	50.64 ± 31.52	7.9	0.145
INR	1.89 ± 1.58	2.16 ± 1.50	17.5	<0.001	2.05 ± 1.43	2.15 ± 1.50	6.6	0.233
Creatinine (mg/dL)	2.01 ± 1.65	1.87 ± 1.52	8.8	0.034	1.94 ± 1.57	1.87 ± 1.52	4.6	0.396
Hemoglobin (g/dL)	9.5 ± 2.04	8.5 ± 1.88	52.5	<0.001	8.61 ± 1.89	8.51 ± 1.87	5.4	0.324
Lactate, *n* (%)			48.0	<0.001			8.5	0.652
(0.2)	2,646 (37.40)	148 (21.70)			152 (22.50)	148 (21.90)		
(2.4)	2,105 (29.70)	198 (29.10)			211 (31.20)	198 (29.20)		
(4.10)	1,289 (18.20)	197 (28.90)			179 (26.40)	196 (29.00)		
>10	173 (2.40)	65 (9.50)			54 (8.00)	63 (9.30)		
Missing value	871 (12.30)	73 (10.70)			81 (12.00)	72 (10.60)		
Infection site, *n* (%)			29.7	<0.001			3.5	0.938
Respiratory	2,728 (38.50)	298 (43.80)			287 (42.40)	297 (43.90)		
Gastrointestinal	814 (11.50)	116 (17.00)			114 (16.80)	115 (17.00)		
Urinary	1,725 (24.40)	94 (13.80)			99 (14.60)	94 (13.90)		
Other	1,817 (25.60)	173 (25.40)			177 (26.10)	171 (25.30)		
qSOFA score	1.99 ± 0.67	1.90 ± 0.61	13.9	0.001	1.89 ± 0.66	1.90 ± 0.61	1.4	0.798
SOFA score	6.84 ± 3.12	8.82 ± 3.61	58.8	<0.001	8.69 ± 3.80	8.81 ± 3.61	3.2	0.553
Treatment								
Renal replacement therapy, *n* (%)	625 (8.80)	62 (9.10)	1.0	0.805	74 (10.90)	61 (9.00)	6.4	0.238
Mechanical ventilation, *n* (%)	3,838 (54.20)	472 (69.30)	31.5	<0.001	460 (67.90)	469 (69.30)	2.9	0.598
Vasopressor use, *n* (%)	2,367 (33.70)	272 (39.90)	12.9	0.001	271 (40.00)	269 (39.70)	0.6	0.912
Antiplatelet drug use, *n* (%)	3,168 (44.70)	189 (27.80)	35.9	<0.001	189 (27.90)	189 (27.90)	0.1	1.000
Warfarin use, *n* (%)	1,468 (20.70)	69 (10.10)	29.6	<0.001	64 (9.50)	69 (10.20)	2.5	0.648

*Results expressed as mean ± standard deviation, n (%), and p-value was generated by T-test or chi-square method between the two groups. Of 7765 patients in the full cohort, 479 (6.2%) had missing information on INR, 2 patients had missing information on hemoglobin. SICU, surgical intensive care unit; MICU, medical intensive care unit; INR, International Normalized Ratio; qSOFA, quick Sequential Organ Failure Assessment; SOFA, Sepsis-related Organ Failure Assessment*.

The primary and secondary outcomes of the propensity-matched analysis are displayed in Table 2. The adjusted 28- [36.9 vs. 30.4%, odds ratio (OR), 1.21; 95% confidence interval (CI), 1.01–1.46; *p* = 0.039] and 90-day (50.8 vs. 44.6%, OR, 1.13; 95% CI, 1.00–1.31; *p* = 0.048) all-cause mortality rates in the platelet transfusion group were higher than those in the no transfusion group. The rates of RBC transfusion (30.7 vs. 26.7%) were not significantly different between the two groups (*p* = 0.149). Regarding other secondary outcomes, the mean number of ICU-free days was significantly lower in the transfusion group than in the non-transfusion group (15.88 ± 8.97 vs. 18.64 ± 8.33 days, *p* <0.001); the mean number of hospital-free days was significantly lower in the transfusion group than in the non-transfusion group (10.29 ± 8.49 vs. 11.43 ± 8.85 days, *p* = 0.017).

**Table 2:** Primary and secondary outcomes in the propensity score matched cohort.

	**Not transfusion (*n* = 677)**	**Transfusion (*n* = 677)**	**OR or mean difference (95%CI)**	***P*** **-value**
**Primary outcome**				
28-day mortality, *n* (%)	206 (30.4)	250 (36.9)	1.21 (1.01, 1.46)	0.039^a^
90-day mortality, *n* (%)	302 (44.6)	344 (50.8)	1.13 (1.00, 1.31)	0.048^a^
**Secondary outcome**				
RBC Transfusion, *n* (%)	181 (26.7)	208 (30.7)	1.15 (0.94, 1.40)	0.149^a^
ICU-free days^c^, mean ± SD	18.64 ± 8.33	15.88 ± 8.97	−2.76 (−3.68 to −1.85)	<0.001^b^
Hospital-free days^c^, mean ± SD	11.43 ± 8.85	10.29 ± 8.49	−1.14 (−2.07 to −0.20)	0.017^b^

a *Estimate and p-value comes from conditional logistic regression analysis*.

b *Estimate and p-value comes from paired student-t test*.

c *ICU-and hospital-free days at Day 28. RBC, red blood cell; OR, odd ratio*.

**Figure 3:**
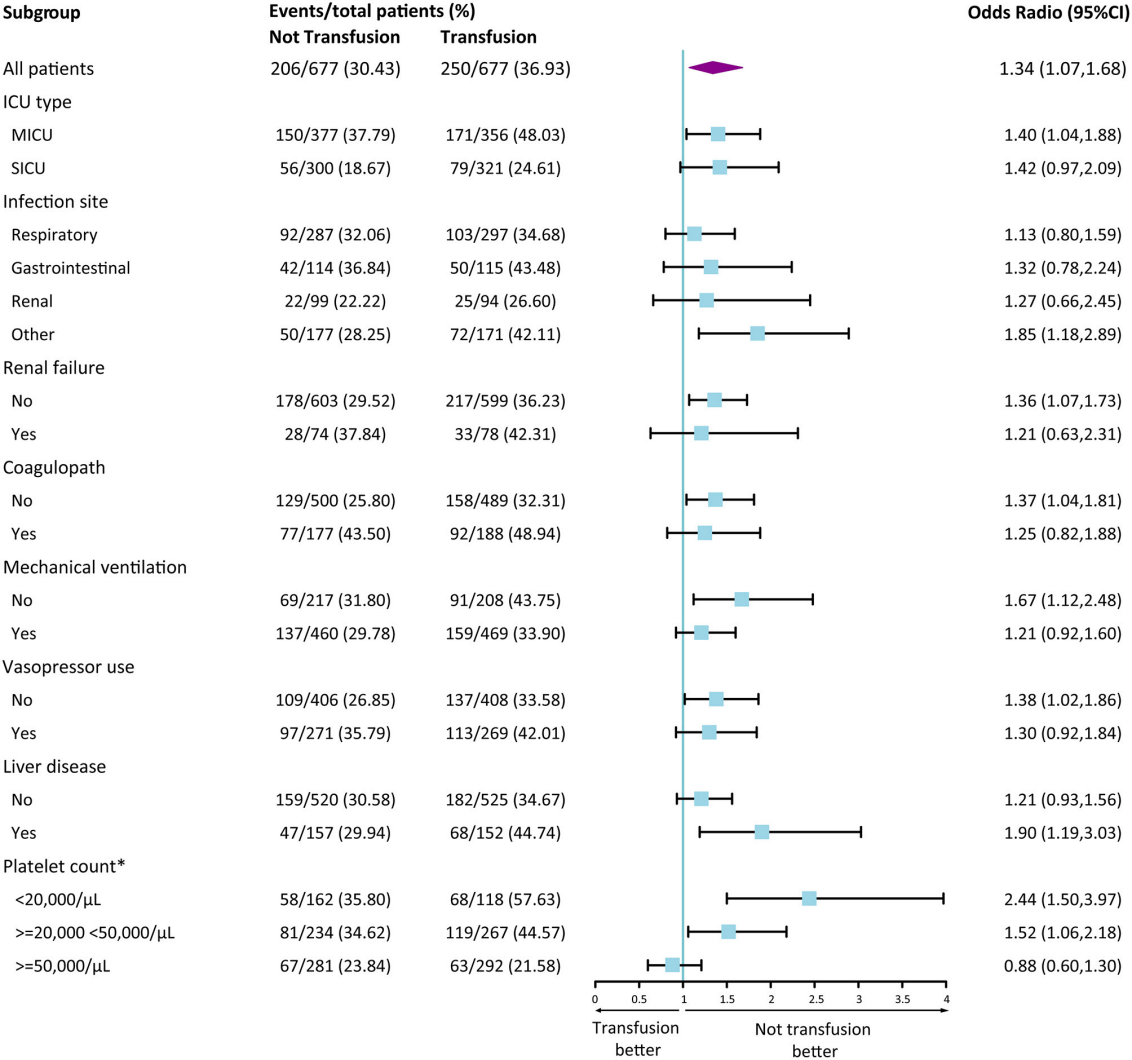
Forest Plot of 28-day mortality according to subgroup in the propensity score matched cohort. Data are shown as the number of 28-day mortality events per total number of patients in that subgroup. Forest plot was used to illustrate the results of multivariate logistic regression analysis according to various subgroups. A Odd Ratio of more than 1.00 indicates a higher risk of death with transfusion group than those with not transfusion. *Interaction analyses were conducted according to platelet group. <20,000/μL vs. 20,000–50,000/μL, P for interaction = 0.155; <20,000/μL vs. ≥50,000/μL, P for interaction <0.001; 20,000–50,000/μL vs. ≥50,000/μL, P for interaction = 0.055. CI, confidence interval.

Figure 3 presents the results of the stratified analysis. Similar results was observed in each subgroup according to ICU type, infection site, renal failure, coagulopathy, mechanical ventilation, vasopressor use, liver disease. In different platelet count group, results showed a different trend (<20,000 vs. ≥50,000/μL, P for interaction <0.001). In addition, the results from the three sensitivity analyses were presented in Supplementary Tables 3–5. It was consistent with our main conclusion that ICU patients with sepsis and thrombocytopenia who did not receive platelet transfusion showed better clinical outcomes.

## Discussion

Results from our study did not show a mortality benefit from platelet transfusion among 7,765 patients with sepsis in the MIMIC-III Critical Care database and in the 677 pairs of patients who were included after propensity score matching. In addition, patients treated with platelet transfusion had fewer ICU- and hospital-free days. Importantly, this study provides valuable evidence for the current sepsis guidelines, which recommends platelet transfusion on the basis of trials in patients with chemotherapy-induced thrombocytopenia (1). This study's findings support that platelet transfusion may not be necessary for patients with sepsis, irrespective of ICU type, infection site, and platelet count.

Platelets play a unique pathophysiological role in the human body, contributing to thrombosis and hemostasis, participating in the inflammatory response, enhancing endothelial barrier function, and promoting tissue regeneration for wound healing (18–20). In sepsis, thrombocytopenia can result from multiple causes, including hypersplenism, bone marrow failure, use of heparin or other drugs, and hemodilution (21, 22). A consensus has been reached that decreased platelet counts are associated with a poor prognosis in patients with sepsis (23). Sepsis-associated coagulopathy, with increased risks of major bleeding and mortality, is characterized by a prolonged prothrombin/INR time and reduced platelet counts (24).

In theory, platelet transfusion can treat thrombocytopenia and improve body function; this would support the argument for a liberal transfusion strategy in patients with thrombocytopenia. At present, study data suggest that the results of platelet transfusion have not been as expected. In the randomized trial by Curley et al. patients who received prophylactic platelet transfusion at higher thresholds (25,000 vs. 50,000/μL) had a higher mortality risk (25). In contrast, a retrospective study in China showed a reduction in mortality in patients with platelet counts between 30,000 and 49,000/μL who received transfusion compared to that in patients with platelet counts <30,000/μL (26). In a multicenter prospective cohort study, the total platelet dose transfused was independently associated with increased ICU morbidity and mortality (27). Our findings were concordant with the prior study which described ICU patients with sepsis-associated thrombocytopenia undergoing platelet transfusion that showed an increase in 28-day all-cause mortality, regardless of the platelet threshold (13).

Most previous research has focused on pediatric, critically ill, surgical, and hematological malignancy patients (11, 25, 26). We noted additional results from a large transfusion registry database, incorporating only a small proportion of sepsis patients and not exploring the effect of platelet transfusion on mortality (28). In a randomized superiority study of 372 patients with dengue fever, prophylactic platelet transfusion demonstrated no advantage over supportive care in preventing bleeding and may have been associated with adverse events (29). Unfortunately, there was no mention of whether the participants met the sepsis criteria. Additional research is needed to assess the effects of platelet transfusion in sepsis patients.

One retrospective study reported increasing platelet counts could improve survival and reduce mortality; however, this intervention used recombinant human thrombopoietin rather than platelet transfusion (5). Our study reached the opposite conclusion in patients who received platelet transfusion. Increasing the platelet count alone may improve the prognosis, but platelet transfusion may not the best method to correct depressed platelet counts (6).

Platelet transfusion not only fail to reduce mortality but also fail to reduce the incidence of bleeding. Two randomized controlled studies found no improvement in bleeding outcomes when thrombocytopenia was corrected before invasive procedures (30, 31). The PLADO trial suggested that platelet transfusion did not change bleeding outcomes even on the day of bleeding (32). Moreover, a retrospective analysis of data collected from a double-blind placebo-controlled trial of patients undergoing coronary artery bypass grafting showed that patients who received platelet transfusion experienced more bleeding events than those who did not receive platelet transfusion (33).

The adverse prognosis due to platelet transfusion must be interpreted with caution, as patients who receive transfusion often have severe disease, and this confounding cannot be eliminated. Currently, increasing evidence relates transfusion to a higher risk of death; possible mechanisms might involve excessive volume load, adult acute respiratory distress syndrome, and transfusion reactions. Additional potential hazards include an increased risk of infection or thrombosis, exacerbation of immune dysfunction, and platelet-monocyte aggregation (9, 10, 12, 13). During sepsis, the coagulation system is activated, and the anticoagulation and fibrinolytic systems are inhibited, which promotes microthrombus formation and leads to microvascular dysfunction (34, 35). In the lower platelet count group, where coagulation activation is more active, platelet transfusion may further exacerbate thrombosis and lead to microvascular occlusion of tissues and organs, resulting in organ damage (36).

This study has several strengths. First, the MIMIC-III dataset contains detailed clinical information, which allowed us to analyse many confounding variables for optimal matching. The second strength is the completeness of the data, with missing values in fewer than 1% of variables. Lactate had the most missing values (12.31%), but we obtained robust results by creating dummy variables and using mean value imputation. Third, the characteristics of the patients and their outcomes were similar to those observed in a larger study involving patients with sepsis in the ICU, suggesting the patients in our dataset are fairly representative of the target ICU population (37).

Several limitations should also be considered when interpreting our results. As with all retrospective studies, our study is subject to unidentified and uncorrected confounding. However, we used propensity score matching, an accessible and valuable tool, to minimize the impact of baseline differences and to balance patients with respect to the possibility of unmeasured confounding. Owing to missing the information of time-dependent covariates in determining platelet transfusion such as bleeding events and coagulation abnormalities, we failed to use time-dependent propensity score method to adjust in this study. Second, we were unable to review the appropriateness of the indication for transfusion since the basis for a physician's decision was not documented. Despite this, we constructed a logistic regression model using platelet transfusion as the dependent variable and obtained a larger area under the curve. Third, the reasons for administering platelet transfusion were unclear. However, we believe that our study population fulfilled the real-world clinical scenario of platelet transfusion in patients with sepsis with thrombocytopenia based on the inclusion and exclusion criteria. Fourthly, this study lacked information regarding the temporal association between platelet transfusion and significant bleeding, resulting in an inability to identify bleeding events objectively. Hence, we selected RBC transfusion as a substitute variable for major bleeding. Finally, since this was a retrospective cohort study, no causation can be shown, and further randomized controlled trials of high quality are needed to examine the role of platelet transfusion in sepsis with thrombocytopenia.

## Conclusion

ICU patients with sepsis and thrombocytopenia who underwent platelet transfusion had higher all-cause mortality at 28- and 90- days and fewer ICU- or hospital-free days than those who did not undergo platelet transfusion. RBC transfusion rates were similar between the two groups. Despite this, high quality randomized controlled trials are needed to validate our ideas.

## Data Availability Statement

The datasets presented in this study can be found in online repositories. The names of the repository/repositories and accession number(s) can be found below: https://mimic.mit.edu/.

## Ethics Statement

Ethical review and approval was not required for the study on human participants in accordance with the local legislation and institutional requirements. Written informed consent for participation was not required for this study in accordance with the national legislation and the institutional requirements.

## Author Contributions

YC conceived of the study, participated in its design and coordination and helped to review the manuscript. SH participated in the design of the study, performed the statistical analysis, and revised the manuscript. CF participated in collection and assembly of data, participated in the statistical analysis, and drafted the manuscript. JM participated in the assembly of data, statistical analysis, and drafted the manuscript. CT participated in collection and assembly of data and drafted the manuscript. All authors read and approved the final manuscript.

## Conflict of Interest

The authors declare that the research was conducted in the absence of any commercial or financial relationships that could be construed as a potential conflict of interest.

## Publisher's Note

All claims expressed in this article are solely those of the authors and do not necessarily represent those of their affiliated organizations, or those of the publisher, the editors and the reviewers. Any product that may be evaluated in this article, or claim that may be made by its manufacturer, is not guaranteed or endorsed by the publisher.
