# Supplementary material for: Platelet Transfusion in Patients With Sepsis and Thrombocytopenia: A Propensity Score-Matched Analysis Using a Large ICU Database
Source: Front Med (Lausanne). 2022 Feb 16;9:830177. doi: 10.3389/fmed.2022.830177 (PMC8888830; doi:10.3389/fmed.2022.830177)
Supplement: Supplementary file 1 [file Data_Sheet_1.DOCX]

Supplementary Material

# Supplementary Figures


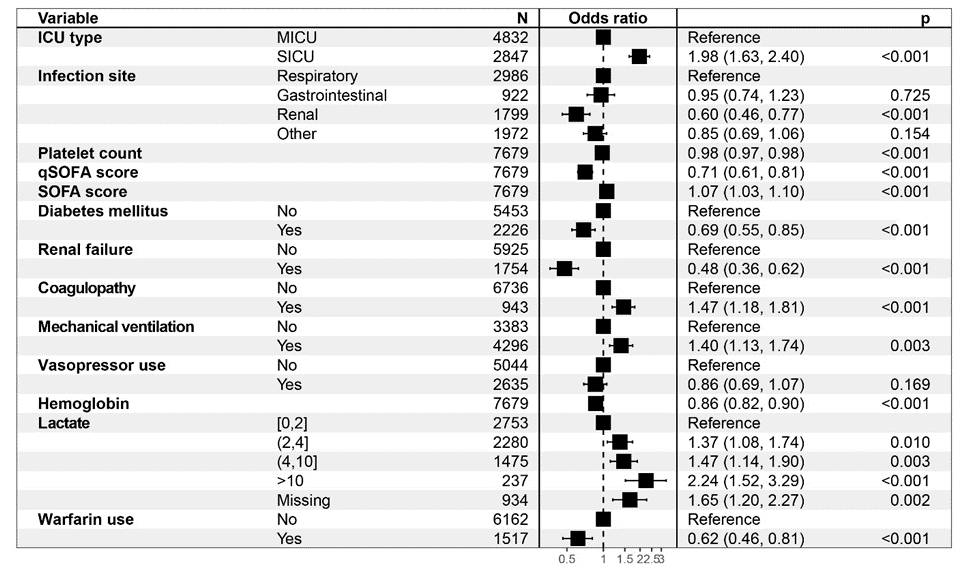


**Supplementary Figure 1.** One-parameter logistic model for propensity score using purposeful selection.

**Notes:** The logistic model was built by ICU type, Infection site, Platelet count, qSOFA score, SOFA score, Diabetes mellitus, Renal failure, Coagulopathy, Mechanical ventilation, Vasopressor use, Hemoglobin, Lactate, Warfarin use, Antiplatelet drug use. **Abbreviations:** SICU, surgical intensive care unit; MICU, medical intensive care unit; qSOFA, quick Sequential Organ Failure Assessment; SOFA, Sepsis-related Organ Failure Assessment.


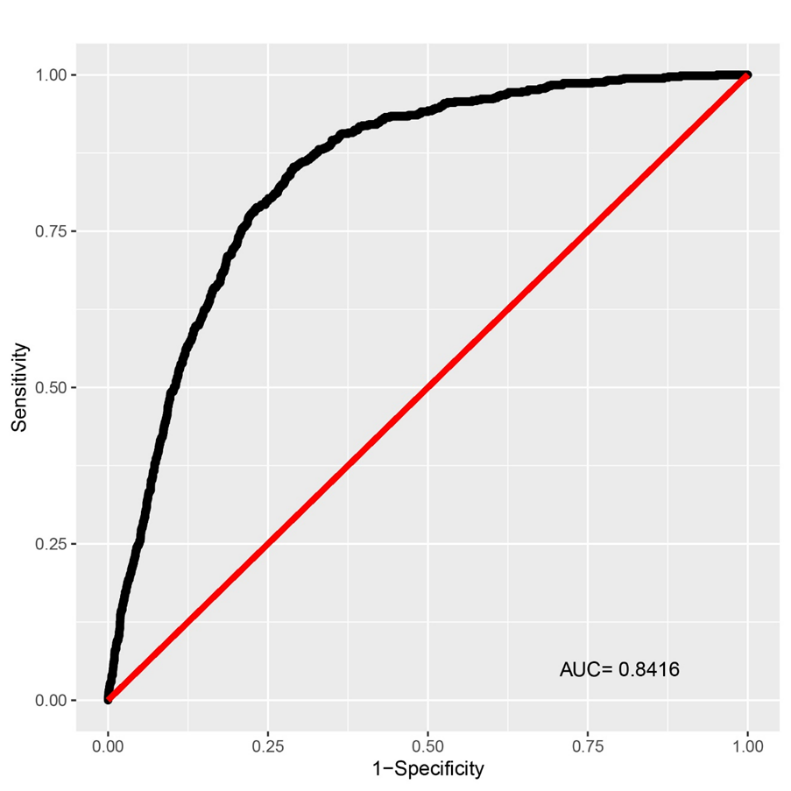


**Supplementary Figure 2.** The receiver operating characteristic curve for predicting platelet transfusion reflecting the discrimination power of the model.

**Notes:** The area under the receiver operating characteristic curve is 0.8416. Generally, the AUC area greater than 0.7 indicates that the model discrimination power is great. **Abbreviations:** AUC, Area under the curve.


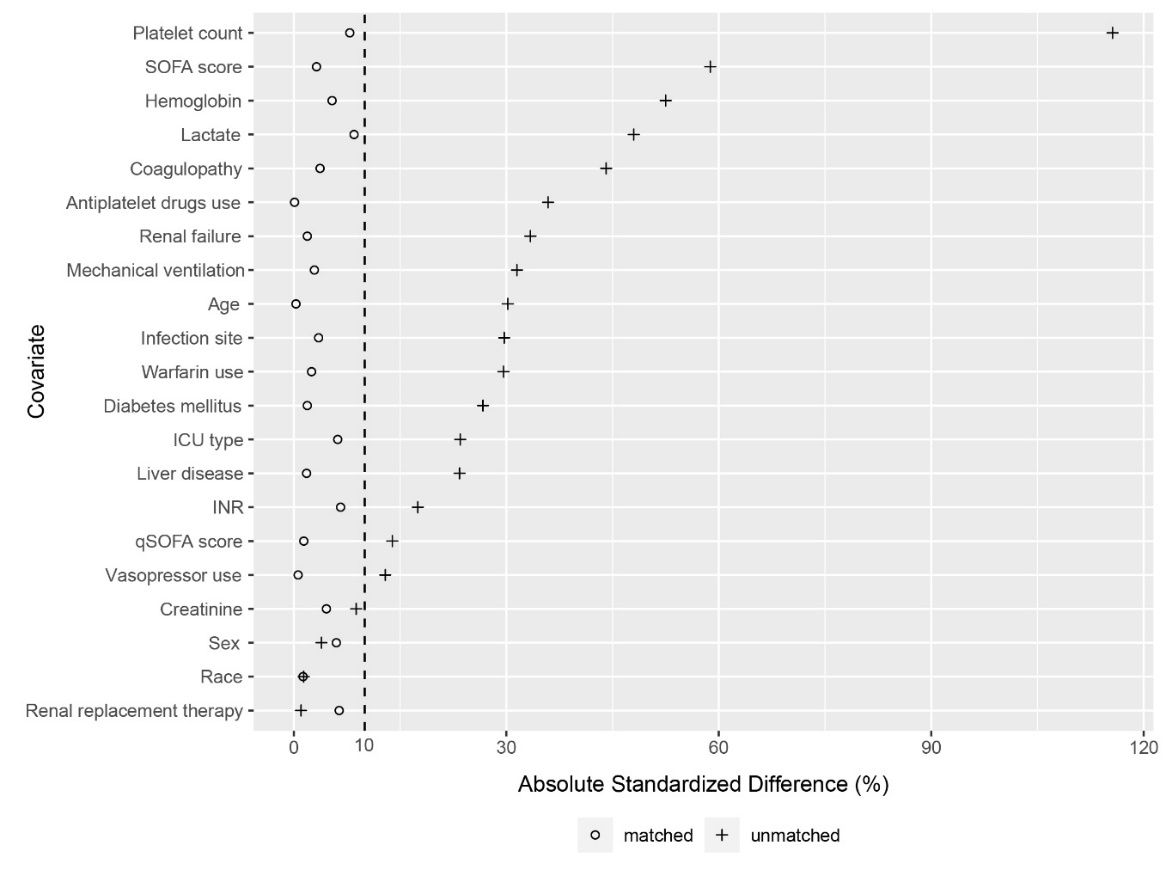


**Supplementary Figure 3.** Standardized mean difference of covariate before and after propensity score matching.

**Notes:** Points to the right of the vertical reference line represent a standardized difference greater than 10.0 between those who received prophylactic platelet transfusion and those who did not. **Abbreviations:** SOFA, Sepsis-related Organ Failure Assessment; INR, International normalized ratio; qSOFA, quick Sequential Organ Failure Assessment.


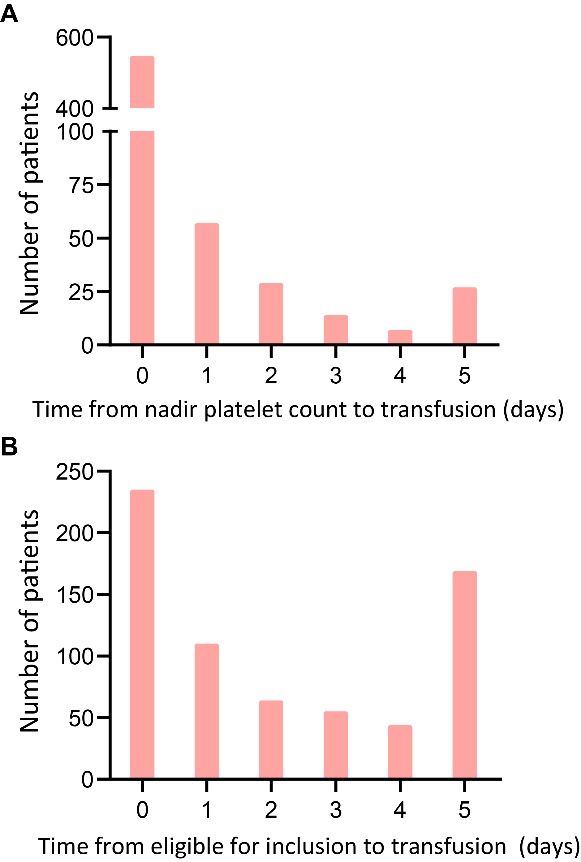


**Supplementary Figure 4.** Number of days from platelet transfusion.

**Notes:** A. Day 0 represents the day of platelet nadir; Day 5 represents 5 and above days from platelet nadir to platelet transfusion. B. Day 0 represents the day of eligible for inclusion; Day 5 represents 5 and above days from eligible for inclusion to platelet transfusion.


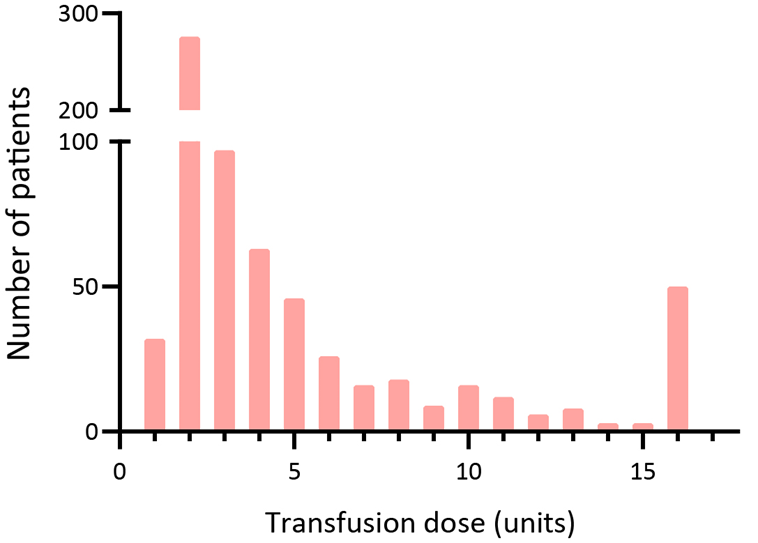


**Supplementary Figure 5.** Number of patients stratified by the total platelet transfusion dose.

**Notes:** The transfusion dose exceeding 16 units is also shown with 16.

# Supplementary Tables

| Covariate | patients with missing lactate (n=944) | patients without missing lactate (n=6821) | SMD（%） | p-value |
| --- | --- | --- | --- | --- |
| Male, n (%) | 536 (56.8) | 3946 (57.9) | 2.2 | 0.556 |
| Age (years) | 62.83±15.87 | 62.02±15.89 | 2.1 | 0.543 |
| White race, n (%) | 658 (69.7) | 4961 (72.7) | 6.7 | 0.056 |
| ICU type, n (%) |  |  |  |  |
| MICU | 321 (34.0) | 2558 (37.5) | 7.3 | 0.040 |
| SICU | 623 (66.0) | 4263 (62.5) | 7.3 | 0.040 |
| Comorbidity |  |  |  |  |
| Diabetes mellitus, n (%) | 248 (26.3) | 2003 (29.4) | 6.9 | 0.054 |
| Renal failure, n (%) | 175 (18.5) | 1607 (23.6) | 12.3 | 0.001 |
| Liver disease, n (%) | 104 (11.0) | 998 (14.6) | 10.8 | 0.003 |
| Coagulopathy, n (%) | 71 (7.5) | 890 (13.0) | 18.3 | <0.001 |
| Laboratory examination |  |  |  |  |
| Platelet count (×1000/μL) | 93.85±39.20 | 87.13±40.27 | 16.9 | <0.001 |
| INR | 1.78±1.17 | 1.93±1.62 | 10.9 | 0.007 |
| Creatinine (mg/dL) | 1.76±1.56 | 2.03±1.65 | 16.7 | <0.001 |
| Hemoglobin (g/dL) | 9.60±2.07 | 9.41±2.04 | 9.2 | 0.008 |
| Infection site, n (%) |  |  | 36.4 | <0.001 |
| Respiratory | 265 (28.1) | 2761 (40.5) |  |  |
| Gastrointestinal | 93 ( 9.9) | 837 (12.3) |  |  |
| Renal | 346 (36.7) | 1473 (21.6) |  |  |
| Other | 240 (25.4) | 1750 (25.7) |  |  |
| qSOFA score | 1.80±0.69 | 2.00±0.66 | 30.6 | <0.001 |
| SOFA score | 5.41±2.17 | 7.23±3.27 | 65.6 | <0.001 |
| Treatment |  |  |  |  |
| Transfusion, n (%) | 73 (7.7) | 608 (8.9) | 4.3 | 0.254 |
| Renal replacement therapy, n (%) | 63 (6.7) | 624 (9.1) | 9.2 | 0.014 |
| Mechanical ventilation, n (%) | 302 (32.0) | 4008 (58.8) | 55.8 | <0.001 |
| Vasopressor use, n (%) | 168 (17.9) | 2471 (36.5) | 42.9 | <0.001 |
| Antiplatelet drug use, n (%) | 386 (40.9) | 2971 (43.6) | 5.4 | 0.130 |
| Warfarin use, n (%) | 190 (20.1) | 1347 (19.7) | 0.9 | 0.818 |

**Supplementary Table 1.** Standardized differences in the patients with missing lactate and patients without missing lactate.

**Notes:** a. Data are mean ± SD or n (%), and p-value was generated by T-test or chi-square method between the two groups. b. INR data were missing for 479 patients (475 in the not transfusion group and 4 in the transfusion group). Hemoglobin data were missing for 2 patients (2 in the transfusion group). **Abbreviations:** SICU, surgical intensive care unit; MICU, medical intensive care unit; INR, International normalized ratio; qSOFA, quick Sequential Organ Failure Assessment; SOFA, Sepsis-related Organ Failure Assessment.

|  | Model inclusion of lactate  (n=7679) | Model without inclusion of lactate  (n=7679) |
| --- | --- | --- |
| (Intercept) | 1.51 (0.85, 2.69) | 1.98 (1.14, 3.45) * |
| SICU | 1.98 (1.63, 2.40) *** | 2.06 (1.70, 2.49) *** |
| Infection site |  |  |
| Respiratory | reference | reference |
| Gastrointestinal | 0.95 (0.74, 1.23) | 1.01 (0.78, 1.30) |
| Renal | 0.60 (0.46, 0.77) *** | 0.62 (0.48, 0.80) *** |
| Other | 0.85 (0.69, 1.06) | 0.89 (0.72, 1.10) |
| Platelet count | 0.98 (0.97, 0.98) *** | 0.98 (0.97, 0.98) *** |
| qSOFA score | 0.71 (0.61, 0.81) *** | 0.69 (0.60, 0.79) *** |
| SOFA score | 1.07 (1.03, 1.10) *** | 1.08 (1.04, 1.11) *** |
| Diabetes mellitus | 0.69 (0.55, 0.85) *** | 0.68 (0.54, 0.84) *** |
| Renal failure | 0.48 (0.37, 0.62) *** | 0.46 (0.35, 0.60) *** |
| Coagulopathy | 1.47 (1.18, 1.81) *** | 1.51 (1.22, 1.87) *** |
| Mechanical ventilation | 1.40 (1.13, 1.74) ** | 1.42 (1.15, 1.76) ** |
| Vasopressor use | 0.86 (0.69, 1.07) | 0.89 (0.72, 1.10) |
| Hemoglobin | 0.86 (0.82, 0.90) *** | 0.85 (0.82, 0.90) *** |
| Lactate |  |  |
| [0,2] | reference |  |
| (2,4] | 1.37 (1.08, 1.74) * |  |
| (4,10] | 1.47 (1.14, 1.90) ** |  |
| >10 | 2.24 (1.52, 3.29) *** |  |
| Missing | 1.65 (1.20, 2.27) ** |  |
| Warfarin use | 0.62 (0.47, 0.81) *** | 0.61 (0.46, 0.81) *** |
| AUC | 0.8416 | 0.8395 |
| AIC | 3642.44 | 3656.84 |
| BIC | 3774.41 | 3761.04 |
| Pseudo R2 | 0.27 | 0.26 |

**Supplementary Table 2.** Differences between propensity scoring models with and without inclusion of lactate.

**Notes:** a. *** p < 0.001; ** p < 0.01; * p < 0.05. b. Pseudo R2 was ranging from 0 to 1 with higher values indicating better model fit. c. Infection site and lactate were entered into the multivariate logistic model as categorical variable (reference group: respiratory and lactate ≤ 2mmol/L, respectively). **Abbreviations:** AUC, the area under the receiver operating characteristic curve; AIC, Akaike information criterion; BIC, Bayesian information criterion.

|  | Not Transfusion (n = 7084) | Transfusion  (n = 681) | OR (95%CI) / Difference (95%CI)^b^ | p-value^a^ |
| --- | --- | --- | --- | --- |
| Primary Outcome |  |  |  |  |
| 28-day mortality, n (%) | 1736 (24.5) | 253 (37.2) | 1.42 (1.17, 1.71) | <0.001 |
| 90-day mortality, n (%) | 2475 (34.9) | 348 (51.1) | 1.40 (1.17, 1.67) | <0.001 |
| Secondary Outcome |  |  |  |  |
| RBC Transfusion, n (%) | 1949 (27.5) | 208 (30.5) | 0.92 (0.76, 1.11) | 0.368 |
| ICU-free days^c^, mean±SD | 20.23±7.63 | 15.91±8.95 | -4.31 (-3.71, -4.93) | <0.001 |
| Hospital-free days^c^, mean±SD | 13.58±8.37 | 10.29±8.49 | -3.29 (-2.63, -3.95) | <0.001 |

**Supplementary Table 3.** Primary and secondary outcomes in the full cohort in which lactate was processed by categorical variables.

**Notes:** a. Estimate and p-value come from logistic regression or negative binomial generalized linear models. b. Adjusted by ICU type, Infection site, Platelet count, qSOFA score, SOFA score, Diabetes mellitus, Renal failure, Coagulopathy, Mechanical ventilation, Vasopressor use, Hemoglobin, Lactate, Warfarin use, Antiplatelet drug use. c. ICU-and hospital-free days at Day 28. **Abbreviations:** RBC, red blood cell; OR, odds ratio.

|  | Not Transfusion (n = 7084) | Transfusion  (n = 681) | OR (95%CI) / Difference (95%CI)^b^ | p-value^a^ |
| --- | --- | --- | --- | --- |
| Primary Outcome |  |  |  |  |
| 28-day mortality, n (%) | 1736 (24.5) | 253 (37.2) | 1.41(1.16, 1.72) | <0.001 |
| 90-day mortality, n (%) | 2475 (34.9) | 348 (51.1) | 1.38(1.15, 1.65) | <0.001 |
| Secondary Outcome |  |  |  |  |
| RBC Transfusion, n (%) | 1949 (27.5) | 208 (30.5) | 0.91(0.74, 1.13) | 0.311 |
| ICU-free days^c^, mean±SD | 20.23±7.63 | 15.91±8.95 | -4.31(-3.71, -4.93) | <0.001 |
| Hospital-free days^c^, mean±SD | 13.58±8.37 | 10.29±8.49 | -3.29(-2.63, -3.95) | <0.001 |

**Supplementary Table 4.** Primary and secondary outcomes in the full cohort in which lactate was processed by conditional mean interpolation.

**Notes:** a. Estimate and p-value come from logistic regression or negative binomial generalized linear models. b. Adjusted by ICU type, Infection site, Platelet count, qSOFA score, SOFA score, Diabetes mellitus, Renal failure, Coagulopathy, Mechanical ventilation, Vasopressor use, Hemoglobin, Lactate, Warfarin use, Antiplatelet drug use. c. ICU-and hospital-free days at Day 28. **Abbreviations:** RBC, red blood cell; OR, odds ratio.

|  | Transfusion over one day (n = 7084) | Transfusion within one day  (n = 547) | OR (95%CI) / Difference (95%CI)^b^ | p-value^a^ |
| --- | --- | --- | --- | --- |
| Primary Outcome |  |  |  |  |
| 28-day mortality, n (%) | 1736 (24.5) | 205 (37.5) | 1.48 (1.21, 1.82) | <0.001 |
| 90-day mortality, n (%) | 2475 (34.9) | 273 (49.9) | 1.38 (1.13, 1.67) | 0.002 |
| Secondary Outcome |  |  |  |  |
| RBC Transfusion, n (%) | 1949 (27.5) | 165 (30.2) | 0.88 (0.71, 1.08) | 0.160 |
| ICU-free days^c^, mean±SD | 20.23±7.63 | 16.12±9.07 | -4.10 (-3.43, -4.78) | <0.001 |
| Hospital-free days^c^, mean±SD | 13.58±8.37 | 10.88±8.61 | -2.70 (-1.98, -3.43) | <0.001 |

**Supplementary Table 5.** Primary and secondary outcomes in the transfusion over one day and within one day.

**Notes:** a. Estimate and p-value come from logistic regression or negative binomial generalized linear models. b. Adjusted by ICU type, Infection site, Platelet count, qSOFA score, SOFA score, Diabetes mellitus, Renal failure, Coagulopathy, Mechanical ventilation, Vasopressor use, Hemoglobin, Lactate, Warfarin use, Antiplatelet drug use. c. ICU-and hospital-free days at Day 28. **Abbreviations:** RBC, red blood cell; OR, odds ratio.
